# Novel Nanoarchitectured Cu_2_Te as a Photocathodes for Photoelectrochemical Water Splitting Applications

**DOI:** 10.3390/nano12183192

**Published:** 2022-09-14

**Authors:** Dong Jin Lee, G. Mohan Kumar, V. Ganesh, Hee Chang Jeon, Deuk Young Kim, Tae Won Kang, P. Ilanchezhiyan

**Affiliations:** 1 Quantum-Functional Semiconductor Research Center (QSRC), Institute of Future Technology, Dongguk University, Seoul 04623, Korea; 2 Department of Physics and Nanotechnology, SRM Institute of Science and Technology, Kattankulathur, Chennai 603203, India; 3 Division of Physics and Semiconductor Science, Dongguk University, Seoul 04623, Korea

**Keywords:** copper telluride, nanorods, nanosheets, photocathodes, PEC water splitting

## Abstract

Designing photocathodes with nanostructures has been considered a promising way to improve the photoelectrochemical (PEC) water splitting activity. Cu_2_Te is one of the promising semiconducting materials for photoelectrochemical water splitting, the performance of Cu_2_Te photocathodes remains poor. In this work, we report the preparation of Cu_2_Te nanorods (NRs) and vertical nanosheets (NSs) assembled film on Cu foil through a vapor phase epitaxy (VPE) technique. The obtained nano architectures as photocathodes toward photoelectrochemical (PEC) performance was tested afterwards for the first time. Optimized Cu_2_Te NRs and NSs photocathodes showed significant photocurrent density up to 0.53 mA cm^−2^ and excellent stability under illumination. Electrochemical impedance spectroscopy and Mott–Schottky analysis were used to analyze in more detail the performance of Cu_2_Te NRs and NSs photocathodes. From these analyses, we propose that Cu_2_Te NRs and NSs photocathodes are potential candidate materials for use in solar water splitting.

## 1. Introduction

Photoelectrochemical (PEC) water splitting has been regarded as attractive technology because it provides sustainable and alternative source of energy [1,2,3,4]. Semiconductor electrodes play a vital role for efficient solar water splitting to produce clean and renewable H_2_ and O_2_ [5,6]. Designing semiconductor photoelectrodes with suitable band edge energies that function as energy converters, photosensitizers, and have good photostability, is of fundamental importance. Silicon showed great promise as a photocathode material due to its band gap and earth abundance. However, it suffers from drawbacks such as high reflectivity, limited photovoltage, sluggish reaction kinetics, and photocorrosion in aqueous solution [7,8,9]. Research from several groups has investigated photocathode materials based on transition metal chalcogenides [10,11], phosphides [12,13], oxides [14,15], Sb_2_Se_3_ [16], CdTe [17], and SnS [18]. However, their performance and chemical stability needs to be improved. It is still a significant challenge in developing an ideal photocathode material with long-term stability and high efficiency for practical applications.

Recently copper chalcogenides have gained considerable attention owing to their wide range of phases and compositions, which allows them to tune their physical and chemical properties for a diverse range of applications [19,20,21,22,23,24]. Among them, copper telluride (Cu_2_Te), a p-type semiconductor has gained considerable interest for its potential application in solar cells [25], memory devices [26], lithium ion batteries [27], and thermoelectric materials [28]. In the past, studies on Cu_2_Te were mainly on their thermoelectric and electrical properties. Applications of Cu_2_Te materials in PEC water splitting have been seldomly explored. For example, Sangeetha et al., studied the electrocatalytic water reduction of Cu_2−x_Te by tuning Cu overvoltage. The catalyst shows a low overpotential of 347 mV at 10 mA cm^−2^ towards the HER [29]. On the other hand, Ghosh et al., reported electrochemically deposited Cu_7_Te_4_ thin films to degrade organic pollutant dyes under visible light irradiation. The Cu_7_Te_4_ thin film showed superior photoactivity to reduce toxic Cr(VI) to Cr(III) and the effective removal of Cr(VI) up to 99.8% in 30 min [30]. More recently, Cu_7_Te_4_ nanosheet was prepared by Wang et al., for OER activity and showed a low overpotential of 323 mV to drive the current density of 10 mA cm^−2^ and a small Tafel slope of 86 mV dec^−1^ [31].

Inspired by these, we report the direct synthesis of Cu_2_Te NRs and vertical NSs on Cu foil via VPE. In addition, there have been no reports on the preparation of one-dimensional (1D) and two-dimensional (2D) like Cu_2_Te (e.g., nanorods and vertical nanosheets) by utilizing Cu foil as both substrate and Cu resource. The structural and morphological properties of as-synthesized Cu_2_Te nanostructures were systematically assessed. The as-prepared Cu_2_Te nanostructured samples were then utilized as the photocathode for studying the PEC properties under illumination for the first time. The photocathode based on vertical NSs exhibits excellent PEC performance compared to NRs. The enhanced performance can be due to a large surface area and more active sites, which can provide a high interfacial contact between the electrolytes for fast interfacial charge transfer.

## 2. Experimental Section

### Preparation of Cu_2_Te Nanostructures

The 1D and 2D Cu_2_Te nanostructures were grown via a VPE system used in previous studies [32,33]. Tellurium (Te) powder as chemical precursor, and Cu foil as the substrate, were chosen for the deposition of Cu_2_Te nanostructures. In the Cu_2_Te growth process, 0.5 g high purity (99.999%) Te source is placed in the source zone of the VPE-chamber and Cu substrate were placed 10 cm away from the Te source in the downstream direction. Before flowing the gas, the initial chamber was evacuated to a vacuum of 2 × 10^−3^ Torr using a rotary pump. The quartz chamber was purged with N_2_ gas (200 sccm) and then sufficiently flowed for 10 min to remove residual oxygen and then carrier N_2_ gas was maintained at 50 sccm. In all growth processes, the growth time was 30 min, the temperature of the source zone was maintained at 700 °C, and the operating pressure at this time was 3.5 × 10^−1^ Torr. The growth time is 30 min. For the production of different nanostructures, the growth temperature of Cu_2_Te was set at 550 °C for nanorods and 600 °C for nanosheets, respectively, and carrier N_2_ gas was maintained at 50 sccm. More details about the characterization techniques and photoelectrochemical measurements are described in the Supplementary Information (SI).

## 3. Results and Discussions

**Figure 1:**
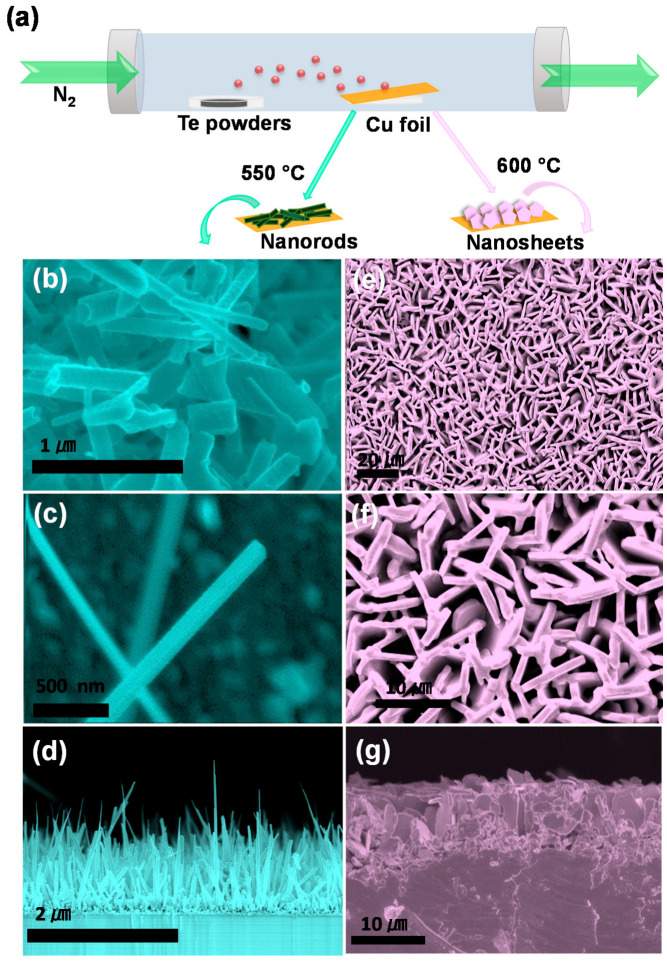
Sample preparation and morphology characterization. (**a**) Schematic of the fabrication process of Cu_2_Te nanostructured film prepared on the Cu foil. (**b**,**c**) SEM and (**d**) cross section image of Cu_2_Te NRs and (**e**,**f**) SEM and (**g**) cross section image of Cu_2_Te NSs.

Cu_2_Te nanostructured film was synthesized via a vapor phase epitaxy (VPE) route from the reaction of Te powder and Cu foil. Figure 1a presents the schematic of the fabrication process of Cu_2_Te nanostructured film prepared on the Cu foil. From the Figure 1, it can be seen that Te powders placed at the source zone of the quartz tube and Cu foil placed at the downstream direction. When the growth temperature is reached, Te powders were evaporated, carried by N_2_ gas will react with the Cu foil substrate at 550 °C and 600 °C to form into Cu_2_Te NRs and vertical NSs films (detailed synthesis processes are provided in the experimental section). The surface morphologies of as deposited Cu_2_Te nanostructured films were studied by FESEM technique. Figure 1b,c displays the SEM images of the as-prepared film on the surface of Cu foil at 550 °C, showing the deposition of numerous NRs on the whole Cu foil. Typical NRs have length of ~1 μm as can be seen in the cross-sectional SEM image (Figure 1d). Similarly, the SEM images (Figure 1e,f) show vertically aligned NSs morphologies on Cu foil deposited at 600 °C with uniform distribution. Moreover, the vertical Cu_2_Te NSs arrays with a thickness of several hundred nanometers randomly extended can be observed from the cross-sectional SEM image in Figure 1g.

**Figure 2:**
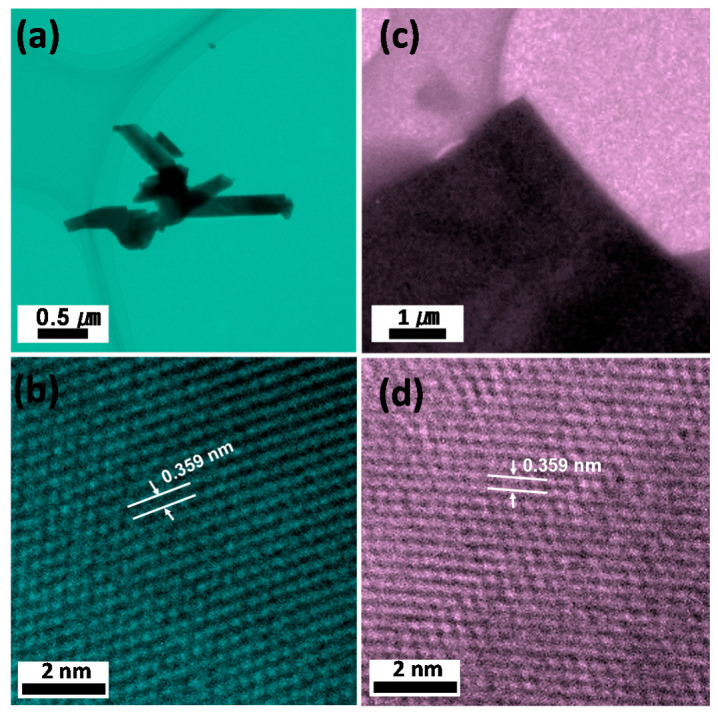
TEM images (**a**,**c**) and HRTEM image (**b**,**d**) of Cu_2_Te NRs and vertical NSs.

The morphologies of Cu_2_Te nanostructured films were further studied by high resolution transmission electron microscopic (HRTEM) techniques as shown in Figure 2. The TEM images in Figure 2a,c further demonstrate the NRs and NSs morphology. The clearly resolved lattice fringe is measured to be about 0.359 nm for both the nanostructures, corresponding well to the (211) plane of Cu_2_Te.

**Figure 3:**
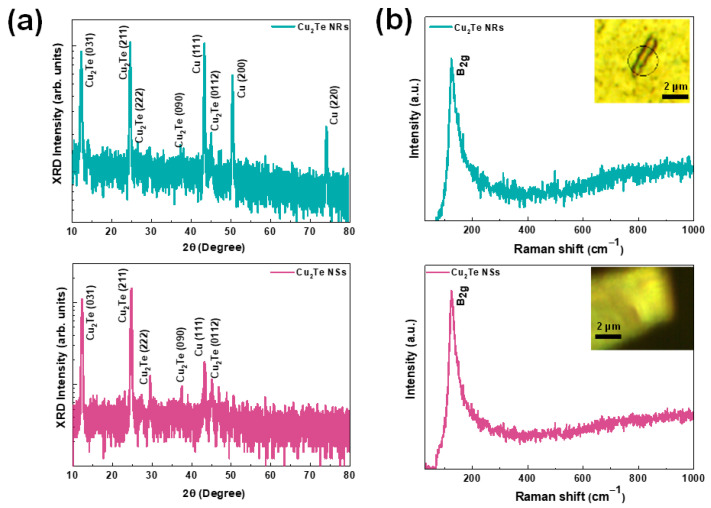
X-ray diffraction pattern of (**a**) Cu_2_Te NRs and vertical NSs and (**b**) Raman spectrum of Cu_2_Te NRs and vertical NSs. Inset shows the optical micrograph image of studied NRs and NSs.

The phase structure of as-synthesized Cu_2_Te NRs and NSs films, were investigated through XRD patterns as depicted in Figure 3a. Here, several diffraction peaks were observed for both Cu_2_Te NRs and NSs films, which can be assigned to orthorhombic Cu_2_Te (PDF # 00-037-1027). The diffraction peaks can be indexed as (031), (211), (222), and (0118) planes of Cu_2_Te. More specifically, both the films show a predominant peak at 24.7°, which indicates that the (211) plane is highly preferred, which is in agreement with HRTEM image. Apart from Cu_2_Te peaks, the two Cu (111) and (200) peaks were seen in the diffraction pattern, which are related to Cu foil.

To further study the structural properties of the Cu_2_Te NRs and vertical NSs, Raman spectroscopy was employed. Figure 3b displays typical Raman spectra of Cu_2_Te NRs and vertical NSs. The image inset shows the optical micrograph image of studied NRs and NSs. Upon 532 nm laser excitation, both the Raman spectrum of NRs and vertical NSs exhibits a sharp peak positioned at 123 cm^−1^, which agrees with the data previously reported for Cu_2_Te [34]. The observed peak has been assigned to B_2g_ vibration mode of Cu_2_Te phase [35,36]. The absence of oxidation-related peaks indicates high quality of the synthesized Cu_2_Te samples.

**Figure 4:**
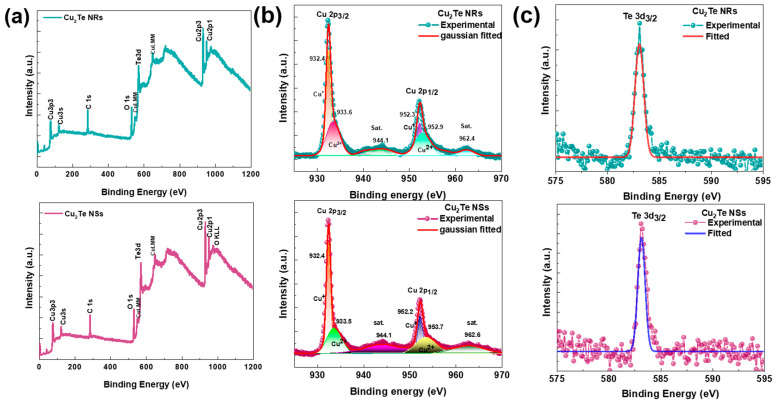
(**a**) Full scan survey spectrum Cu_2_Te NRs and NSs. (**b**) high-resolution spectra of Cu 2p and (**c**) Te 3d of Cu_2_Te NRs and NSs.

X-ray photoelectron spectroscopy (XPS) was adopted to provide information about the chemical bonding and elemental composition in as-grown Cu_2_Te NRs and NSs films. The full survey XPS scan of Cu_2_Te NRs and vertical NSs with element denotation are shown in Figure 4a. The XPS core level spectra of Cu 2p of NRs and NSs films (Figure 4b) show two strong peaks from Cu 2p_1/2_ and Cu 2p_3/2_. As represented by Gaussian fitting, the core level spectra of Cu 2p confirm the presence of Cu^1+^ and Cu^2+^ in both NRs and vertical NSs. The peaks appeared at 932.4 eV (Cu 2p_3/2_) and 952.3 eV (Cu 2p_1/2_) are assigned to Cu^1+^. Similarly, the peaks at 933.6 eV (Cu 2p_3/2_) and 953.7 eV (Cu 2p_3/2_) are the typical values for Cu^2+^, of Cu_2_Te, respectively [37,38]. Additionally, two weak satellite features could be observed at 944.1 eV and 962.4 eV, respectively, and is also attributed to Cu^2+^. This is possibly attributed to the unavoidable surface oxidation under air atmosphere. The XPS core level spectra of Te (Figure 4c) in both NRs and vertical NSs have peaks at binding energies of 583.1 eV, corresponding to Te^2−^ states of Cu_2_Te, respectively [39].

Furthermore, the tellurium-to-copper stoichiometric ratio of Te and Cu of approximately 1:2 is obtained for both the NRs and NSs from the XPS, suggesting grown Cu_2_Te is chemically stoichiometric. The results above demonstrate the successful synthesis of Cu_2_Te on a copper foil.

**Figure 5:**
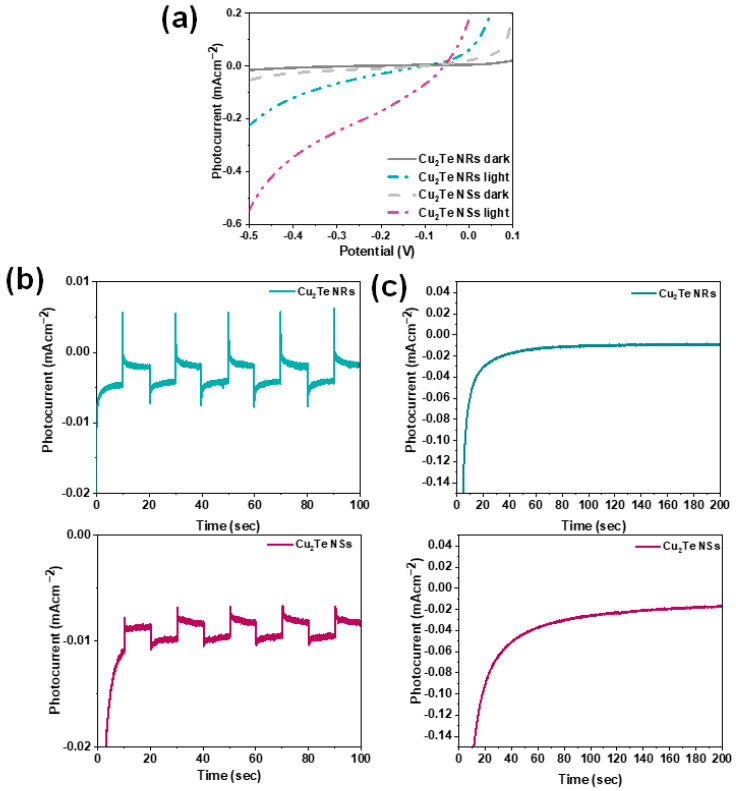
(**a**) Photocurrent density versus voltage curves of Cu_2_Te NRs vertical NSs photocathodes. (**b**) Photocurrent density versus time (I–t) curves of Cu_2_Te NRs and vertical NSs. (**c**) Photostability curve of the Cu_2_Te NRs and vertical NSs.

The PEC performances of the Cu_2_Te nanostructured photocathodes were acquired by linear sweep voltammetry (LSV) measurements under dark and light conditions (100 mW cm^−2^) in 0.5 M Na_2_SO_4_ electrolyte. The photocurrent-density variations for both Cu_2_Te NRs and NSs photocathodes are illustrated in Figure 5a. As observed from Figure 5a, both the samples reveal obvious photocurrent under illumination, demonstrating the efficient charge transfer process at semiconductor/electrolyte interfaces. Besides, the photocurrent density increases with increasing applied potential in the negative direction, implying the p-type conductivity and could possibly be employed as a photocathode material for water splitting. At a bias potential of −0.5 V, the Cu_2_Te NRs photocathodes generate a photocurrent density of 0.21 mA cm^−2^. Interestingly, the photocurrent density of Cu_2_Te vertical NSs photocathodes reached the maximum value of 0.53 mA cm^−2^. The enhanced photocurrent density of the vertical NSs can be ascribed to light absorption utilization via multiple reflections of this vertical structure as well as forming intimate contact with the Cu substrate. Moreover, this vertical NSs architecture with an open morphology, provides more active sites, which can provide a high interfacial contact between the electrolytes for fast interfacial charge transfer, thus improving the PEC performance. According to the reported literature, the observed photocurrent density was quite high (Table S1). The generated maximum photocurrent density was ~2-times higher compared to that of NRs. However, in this study, the photocurrent value of the photocathodes is closely related to the morphological properties of the films.

The chronoamperometric I-t curves examining the photocurrent of the Cu_2_Te NRs and vertical NSs photocathodes under chopped illuminations are shown in Figure 5b. The photocurrent of both NRs and NSs exhibits a prompt rise under each illumination, and quickly drops when the light is turned off, demonstrating the excellent switching behavior and good stability of the photocathodes. Afterward, a photostability test for the Cu_2_Te NRs and vertical NSs (Figure 5c) photocathodes was carried out under continuous illumination and displayed a good stability. Both the photocathodes nearly maintained 70% retention of initial value after 200 s suggesting the excellent stability. Finally, to give more evidence, we also performed XRD measurements for the photocathodes after the PEC stability test. As can be observed from Figure S1, it is worth mentioning that predominant peak at 24.7° appears in the XRD pattern of both Cu_2_Te NRs and vertical NSs photocathodes after the stability test demonstrates its excellent structural stabilities. The above results highlight the promising potential of the Cu_2_Te NSs and NRs as state-of-the art photocathodes.

**Figure 6:**
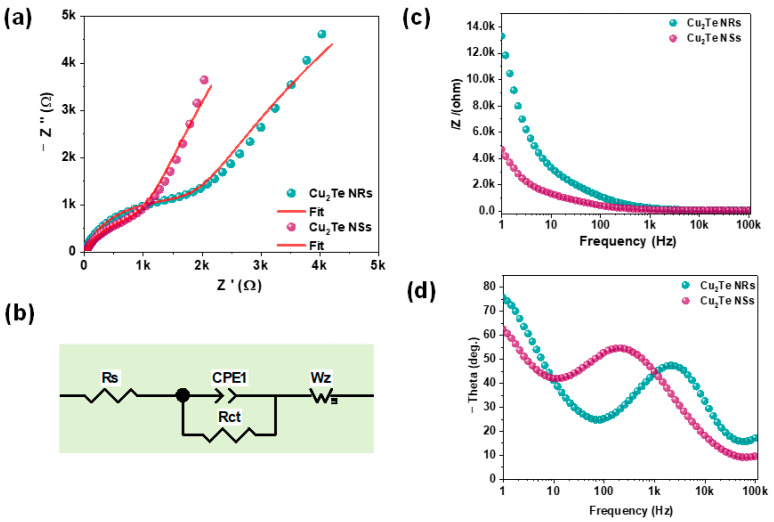
(**a**) Nyquist plots of the Cu_2_Te NRs and vertical NSs. (**b**) Shows the fitted equivalent circuit. (**c**) Bode and (**d**) Phase angle plots of Cu_2_Te NRs and NSs.

The PEC performance of Cu_2_Te NRs and vertical NSs photocathodes was further studied with (EIS) under light illumination. This investigation was made in order to elucidate the charge transfer resistance at the photocathode/solution interface. Figure 6a presents the typical Nyquist curves of Cu_2_Te NRs and vertical NSs photocathodes under illumination. As seen from Figure 6a, both the Nyquist curves show a semicircle arc at high frequency can be used to identify charge transfer resistance and a straight line at low-frequency regions (mass transfer). The fitted equivalent circuit model for the photocathodes is given in Figure 6b, where R_ct_, R_s,_ CPE, and W_Z_ represents charge transfer resistance, electrolyte resistance, constant phase element, and Warburg impedance. The values obtained from the fitted circuit are summarized in Table S2. The charge transfer resistance from Cu_2_Te NSs were found to be systematically decreased as compared with Cu_2_Te NRs. These results suggest that the Cu_2_Te NSs could offer smoother carrier diffusion paths and, hence, higher photoelectrochemical performances compared with the Cu_2_Te NRs. The PEC behavior of the photocathodes was then examined with the log |Z| vs. log frequency (log f) plots in Figure 6c. The vertical NSs photocathodes display minimum |Z| compared to the NRs photocathodes indicating its higher PEC activity. Figure 6d reveals the phase angle vs. log f plots. Here, the Cu_2_Te NRs photocathodes showed a high phase angle around (−75°), while NSs photocathodes exhibits phase angle around (−60°). The less-negative phase angle for the Cu_2_Te NSs in comparison to NRs photocathodes further confirmed a lower resistance to charge mobility in the semiconductor and at the electrolyte/photocathode interface. Additionally, the characteristic peak frequency shifted to a low value for NSs photocathode, indicating it has longer carrier lifetime than in NRs photocathode and thus a lower recombination rate.

**Figure 7:**
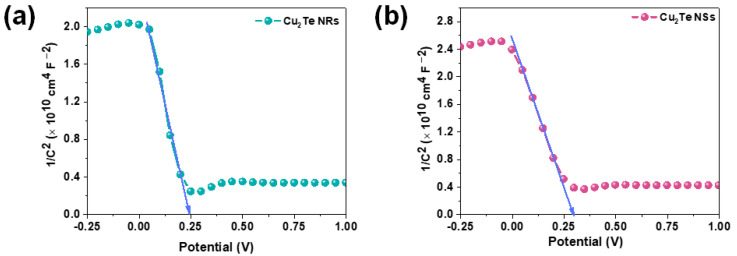
Mott–Schottky plot of (**a**) Cu_2_Te NRs and (**b**) vertical NSs.

Finally, Mott–Schottky (M-S) analysis was employed to estimate charge carrier density (N_A_) and flat band potential (V_fb_) of the Cu_2_Te NRs and NSs photocathodes. Figure 7a,b illustrates the Mott–Schottky plots (1/C^2^ as a function of applied potential) measured at a frequency of 1000 Hz. The negative slope of the plots indicated that both Cu_2_Te NRs and vertical NSs are p-type semiconductors. Furthermore, the flat band potentials of Cu_2_Te NRs and vertical NSs was determined to be 0.25 V and 0.28 V by extrapolating the X intercepts in Mott–Schottky plots. Additionally, the carrier density N_A_ was estimated from Figure 7 using the following equation [40]:

N_A_ = (2/eεε_0_) (d (1/C^2^)/dV)^−1^

where e is the elemental charge, ε the dielectric constant of Cu_2_Te (taken as 17) [41], ε_0_ the permittivity of vacuum, N_A_ is the concentration of charge carriers, and C is the space charge layer capacitance. According to the equation and Figure 7, the N_A_ value of the Cu_2_Te NRs and NSs were determined to be 3.35 × 10^18^ and 4.3 × 10^18^ cm^−3^. Evidently, the higher carrier density in the case of vertical NSs and charge transfer efficiency have contributed to the enhanced PEC performance.

## 4. Conclusions

In summary, we presented a synthesis of Cu_2_Te NRs and vertical NSs assembled film on Cu foil as photocathodes in a water splitting PEC. The morphological and microstructural properties and chemical states of the Cu_2_Te were systematically discussed. It is worth mentioning that the Cu_2_Te NRs and vertical NSs delivered unique photoelectrochemical performances. Compared with NRs, vertical NSs photocathodes exhibited higher photocurrent density. EIS results revealed excellent performance of Cu_2_Te NSs photocathodes, which can be ascribed to low charge transfer resistances across electrolyte/electrode interfaces. We believe that the above results reported herein will open a new avenue in the development of Cu_2_Te-based photocathodes for efficient PEC water splitting.

## Data Availability

Not applicable.

## Supplementary Materials

The following supporting information can be downloaded at: https://www.mdpi.com/article/10.3390/nano12183192/s1, Figure S1: X-ray diffraction pattern of Cu_2_Te NRs and vertical NSs after stability test; Table S1: A comparisons of PEC performances of Cu_2_Te photocathodes is given below; Table S2: Various parameters extracted from the equivalent circuit fit to the EIS data for Cu_2_Te NRs and NSs photocathodes in 0.5M Na_2_SO_4_ electrolyte solutions [42,43,44,45,46].
