# Supplementary material for: Novel Nanoarchitectured Cu2Te as a Photocathodes for Photoelectrochemical Water Splitting Applications
Source: Nanomaterials (Basel). 2022 Sep 14;12(18):3192. doi: 10.3390/nano12183192 (PMC9506189; doi:10.3390/nano12183192)
Supplement: Supplementary file 1 [file nanomaterials-12-03192-s001.zip › nanomaterials-1872374-supplementary.pdf]

nanomaterials

Supplementary Information

Novel Nanoarchitectured Cu_2Te as a Photocathodes for Photoelectrochemical Water Splitting Applications

Dong Jin Lee [1], G. Mohan Kumar [1], V. Ganesh [2], Hee Chang Jeon [1], Deuk Young Kim [1,3], Tae Won Kang [1] and P. Ilanchezhiyan [1,*]

[1] Quantum-Functional Semiconductor Research Center (QSRC), Institute of Future Technology, Dongguk University—Seoul, 04623 Seoul, Korea
[2] Department of Physics and Nanotechnology, SRM Institute of Science and Technology, Kattankulathur, Chennai 603203, India
[3] Division of Physics and Semiconductor Science, Dongguk University—Seoul, 04623 Seoul, Korea
* Correspondence: ilancheziyan@dongguk.edu

1. Characterizations

The morphological characteristics of Cu_2Te samples are analyzed by scanning electron microscopy (SEM) (JEOL JSM-6380, operated at 15 kV) and high-resolution transmission electron microscopy (HRTEM). The phase purity and crystal structure of Cu_2Te NRs and vertical NSs was inferred through Panalytical XPert pro X-ray diffractometer with Cu–Kα radiation (40 kV, 30 mA, λ=1.5418 Å). The Raman measurements were performed in a micro-Raman spectrometer using an excitation wavelength of 532 nm. The chemical composition of the Cu_2Te NRs and vertical NSs was obtained using X-ray photoelectron spectroscopy (XPS-PHI 660).

2. Photoelectrochemical studies

The photoelectrochemical properties Cu_2Te NRs and vertical NSs photocathodes were measured using CHI 200 potentiostat using a three electrode configuration. The VPE grown Cu_2Te NRs and vertical NSs on Cu foil were used as working electrodes, with Ag/AgCl and Pt as reference and counter electrode. All tests were measured in a 0.5 M Na_2SO_4 electrolyte. The photoresponse was measured under a continuous irradiation from a 300 W Xe lamp, and the visible light intensity were calibrated to 100 mW/cm^2 for the incidence onto the photocathode. The area of the photocathode exposed to light was 0.5 cm^2. The photoelectrochemical beaker was positioned 15 cm away from the xenon lamp. Photocurrent stability tests were carried out by measuring the photocurrent under chopped light irradiation at a fixed potential of 0 V vs. RHE. Before PEC measurement, the electrolyte was continuously bubbled with N_2 to remove oxygen and thus eliminate erroneous signals arising from oxygen reduction. Electrochemical impedance spectra (EIS) were measured with the frequency range from 0.1 Hz to 100 kHz under illumination. Mott–Schottky plots were acquired by sweeping the potential at fixed frequencies. Bode phase plots were measured in a frequency range between 0.1 Hz to 100 kHz at open-circuit voltage.

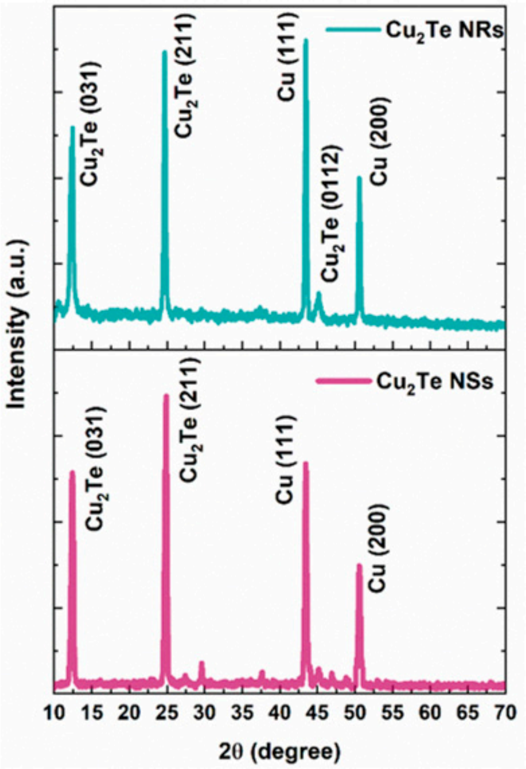

Figure S1. X-ray diffraction pattern of Cu_2Te NRs and vertical NSs after stability test.

Table S1. A comparisons of PEC performances of Cu_2Te photocathodes is given below.

Cu Based Photocathodes	Electrolyte	Maximum Photocurrents (mA cm^{-2})	Reference
$Cu_{2-x}Te$/Cu layers	0.1 M $NaNO_3$	0.014	[1]
Cu_2O/Cu foil	0.2 M K_2HPO_4	0.47	[2]
Cu_2O-CuO	0.5 M K_2SO_4	0.36	[3]
CuO-Cu_2O	0.1 M Na_2SO_4	0.50	[4]
Cu-W Oxide	pH 11 NaOH	0.0010	[5]
Cu_2Te NSs/Cu foil	0.5 M Na_2SO_4	0.53	This work

Table S2. Various parameters extracted from the equivalent circuit fit to the EIS data for Cu_2Te NRs and NSs photocathodes in 0.5M Na_2SO_4 electrolyte solutions.

Samples	R_s (Ω)	R_{ct} (Ω)	W_z (Ω)	C_μ (µF)	$\chi^2 \times 10^{-4}$
Cu_2Te NSs	33.74	1075	0.38	15.47	5.8
Cu_2Te NRs	21.65	2673	0.12	39.11	3.8

References

1. Caballero-Briones, F.; Palacios-Padrós, A.; Peña, J.L.; Fausto Sanz. Phase tailored, potentiodynamically grown p-Cu2-xTe/Cu layers. *Electrochem. commun.* **2008**, *10*, 1684–1687.
2. Dai, P.; Li, W.; Xie, J.; He, Y.; Thorne, J.; McMahon, G.; Zhan, J.; and Wang, D. Forming Buried Junctions to Enhance the Photovoltage Generated by Cuprous Oxide in Aqueous Solutions. *Angew. Chem. Int. Ed.* **2014**, *53*, 13493–13497.
3. Wang, P.; Ng, Y.H.; Amal, R. Embedment of anodized p-type Cu2O thin films with CuO nanowires for improvement in photoelectrochemical stability. *Nanoscale*. **2013**, *5*, 2952–2958.
4. Kim, S.; Kim, H. Cu_2O Thin Film Photoelectrode Embedded with CuO Nanorods for Photoelectrochemical Water Oxidation. *J. Korean Inst. Surf. Eng.* **2019**, *5*, 258-264.
5. Chen, L.; Shet, S.; Tang, H.; Wang, H.; Deutsch, T.; Yan, Y.; Turner, J.; Al-Jassim, M. Electrochemical deposition of copper oxide nanowires for photoelectrochemical applications. *J. Mater. Chem.* **2010,** *20*, 6962–6967.
